# Heterogeneous impacts of home-gardening on household food and nutrition security in Rwanda

**DOI:** 10.1007/s12571-023-01344-w

**Published:** 2023-02-18

**Authors:** Gazali Issahaku, Lukas Kornher, Abu Hayat Md. Saiful Islam, Awal Abdul-Rahaman

**Affiliations:** 1 Center for Development Research, University of Bonn, Bonn, Germany; 2 Department of Food Security and Climate Change, University for Development Studies, Tamale, Ghana; 3 Department of Agricultural Economics, Bangladesh Agricultural University, Mymensingh, Bangladesh; 4 Department of Agribusiness, University for Development Studies, Tamale, Ghana

**Keywords:** Home-gardening, Food and nutrition security, Agricultural commercialization, Impact assessment, Rwanda

## Abstract

This study examines farmers’ decisions to engage in subsistence home-gardening and its impact on food and nutrition security among farm households in Rwanda under diverse conditions. The study uses a nationally representative dataset from Rwanda from 2012, 2015, and 2018. We employ an endogenous switching regression model to jointly estimate the drivers of home-gardening participation decisions and food and nutrition security outcomes, whiles accounting for selection bias from observable and unobservable factors. We also estimate the treatment effects of home-gardening participation on dietary diversity, food consumption score, and anthropometric markers of women and children. The treatment effects are calculated at sample means and connected to market-related variables, such as land ownership, commercialization extent, and market distance. We find that having a home-garden is linked to improved dietary diversity and better nutritional outcomes. If households have restricted access to land and reside further away from marketplaces, the benefits are larger. In contrast, the benefits of home-gardening are positive and significant regardless of the level of commercialization of production. We also discover that family size, gender, education, access to land, and livestock ownership are statistically significant drivers of home-gardening participation in Rwanda. However, the amount of commercialization did not affect a household’s decision to participate in home-gardening.

**Supplementary Information:**

The online version contains supplementary material available at 10.1007/s12571-023-01344-w.

## Introduction

Research has shown that more than two billion people currently suffer from different forms of micronutrient deficiencies, because they do not have access to healthy and diverse diets (Food and Agriculture Organization [FAO] et al., 2021). This figure is likely to increase due to the COVID-19 pandemic. Micronutrient deficiencies can have severe health impacts with long-term consequences on the well-being of children exposed to unhealthy diets in early life (Bailey et al., 2018; FAO et al., 2021). A large share of the malnourished population is smallholder farmers as reported by the International Federation of Red Cross and Red Crescent Societies (2011). For a long time, the following has been the narrative in the economics literature in the Smithsonian spirit: agricultural specialization and commercialization promote growth and lead to welfare and nutrition improvement. However, this view has been challenged, at least as the axiomatic principle (Carletto et al., 2017; Poole et al., 2013). In addition, it was replaced by a more nuanced perspective that considers the theory of agricultural commercialization and nutrition conceptualizing the household decision to move from subsistence to market production (Barrett, 2008; Pingali, 1997; von Braun, 1995). The view also includes the implications for nutrition through changes in income, the availability of home-produced foods, and gender roles within the household (von Braun & Kennedy, 1994). For instance, given the market risks related to specialized agricultural production (mono-cropping), several studies show that off-farm and on-farm diversification are associated with higher dietary diversity and better nutrition outcomes (Zanello et al., 2019; Di Falco & Chavas, 2009; Jones et al., 2014; Islam et al., 2018; Sibhatu et al., 2018; Muthini et al., 2020). As a result, agricultural commercialization and subsistence farming must not always be considered competing concepts; however, they can, in combination, contribute to achieving the Sustainable Development Goals 2 (SDG 2), which is aimed at achieving zero hunger.

To address market risks and to strengthen the local food production, we have identified home-gardens (small home-adjunct plots primarily for vegetable production) as complementary subsistence agriculture to be an effective strategy to increase the availability of nutritious foods (Galhena et al., 2013; Ruel & Alderman, 2013; Weinberger, 2013; Rybak et al., 2018; Schreinemachers et al., 2016; Bahta et al., 2018; Rahmmohan et al., 2019; Castaneda-Navarrero 2021). Studies on home-gardening are often based on case studies looking at home garden interventions coupled with training programs (Marek et al., 1990; Olney et al., 2009; Schreinemachers et al., 2016; Bahta et al., 2018; Baliki et al., 2019; Depenbusch et al., 2021). Except the works of Bahta et al. (2018) and Depenbusch et al. (2021), few studies provide causal relationship between home-gardening and food security. In addition, the study by Bahta et al. (2018) indicates that participation in home-gardening program significantly reduced food insecurity among rural households in South Africa. However, the study by Depenbusch et al. (2021) did not find any causal relationship between home-gardening and food security, suggesting the need for further empirical investigation into this subject. Furthermore, the nature and size of land acquisition and use are issues that affect agriculture in general and therefore, understanding the heterogeneous effects of participating in home-gardening based on land size might influence agricultural policy as well.

The main objective of this study is to examine the heterogeneous impacts of home-gardening on food security among households in Rwanda. Specifically, the study addresses the following objectives as contributions to the empirical literature. First, the study links the home-gardening literature to the theory of agricultural commercialization, which is often overlooked as a driver of home-gardening participation. Second, we examine, how market access, resource endowment, and market risks relate to the food security benefits of home-gardening. In addition, we consider the interaction between home-gardening and agricultural commercialization, and how this affects household food and nutrition security. For instance, the study by Abdoellah et al. (2020) shows a strong and positive correlation between home-gardening and commercialization among farm households in Indonesia. They however, opine that as markets and policies drive the commercialization of food and farming systems, it becomes more difficult for smallholder farmers to maintain home-garden plots. In this study, we define commercialization as the proportion of harvested crops sold. The study tries to examine the food and nutrition security effect of home-gardening participation regarding different levels of commercialization. The study employs an endogenous switching regression (ESR) model (Bahta et al., 2018; Lokshin & Sajaia, 2004) to assess the drivers of home-gardening participation decisions, and their related impacts on food and nutrition security in Rwanda. The ESR model accounts for selection bias from observable and unobservable farm and household factors.

This study uses three rounds of nationally representative household survey data from Rwanda collected by the World Food Program (WFP) and the National Institute of Statistics of Rwanda (NISR) between March and May, a period in which food purchases are high, in 2012, 2015, and 2018. The survey encompasses all 30 districts of Rwanda with data from several indicators of dietary quality and nutrition at the household and individual levels, and with district-level food prices from WFP’s Vulnerability, Analysis, and Mapping (VAM) database. This study tests the following hypothesis: ${H}_{o}:$ Participation in home-gardening has no significant effect on food and nutrition security; ${H}_{1}:$ Participation in home-gardening exerts significant effect on food and nutrition security. Failure to reject the null hypothesis implies that home-gardening does not impact farm households’ food and nutrition security in Rwanda.

The remainder of the article is structured as follows. Section 2 presents a conceptual framework and literature review. Section 3 discusses the study context. Section 4 presents the empirical strategy and analytical framework. Section 5 presents and discusses the empirical results. Section 6 provides the implications of our results for food security and nutrition policies and programs for developing countries. Finally, Sect. 7 concludes the paper.

## Conceptual framework and literature review

Home-gardening has a century-long tradition under both moderate climatic conditions and in tropical and sub-tropical areas. The agricultural literature describes several farming systems – home-gardens, homestead farms, kitchen gardens, urban agriculture, etc. – that overlap and relate to home-gardening. Kumar and Nair (2004) describe home-gardening as the oldest land use activity together with shifting cultivation and provide a general definition of home-gardens as “intimate, multi-story combinations of various trees and crops, sometimes in association with domestic animals, around homesteads” (p. 135). This study closely follows a more restrictive definition by Michelle and Hanstad (2004). We define home-gardens as a small plot area located close to the household’s premises containing a high plant diversity, fully or partially committed for vegetables (Kumar & Nair, 2004) and mainly cultivated using family labor (Ferdous et al., 2016). Home-garden production is primarily for auto-consumption and only supplementary to other sources of family consumption.

Previous studies, except for Depenbusch et al. (2021), have shown a positive association between home-gardening and dietary diversity of all household members, particularly women and children (Galhena et al., 2013; Schreinemachers et al., 2015, 2016; Bahta et al., 2018; Baliki et al., 2019; Rammohan et al., 2019; Castaneda-Navarrero, 2021). Yet, the empirical evidence on the associations with nutritional status remains limited (Masset et al., 2012; Ruel and Alderman, 2013). These results have been documented for various contexts in Africa, South Asia, and Latin America. Moreover, these studies emphasize the role of home-gardening in improving the food environment by increasing availability and access to nutrient-rich foods, particularly vegetables. In addition, home-gardening increases food sovereignty by providing stable access to fresh and nutritious food, particularly in times of scarcity in the market (Abdoelllah et al., 2020). Galhena et al. (2013) emphasize that resource-poor families could benefit more from home-gardening than households that have access to land and capital, particularly if they cannot afford expensive nutritious foods (e.g., animal products) to fulfill their nutritional needs. In line with this, Rammohan et al. (2019) observe that home-gardening improves food security and dietary diversity among the landless households in Myanmar.

**Fig. 1:**
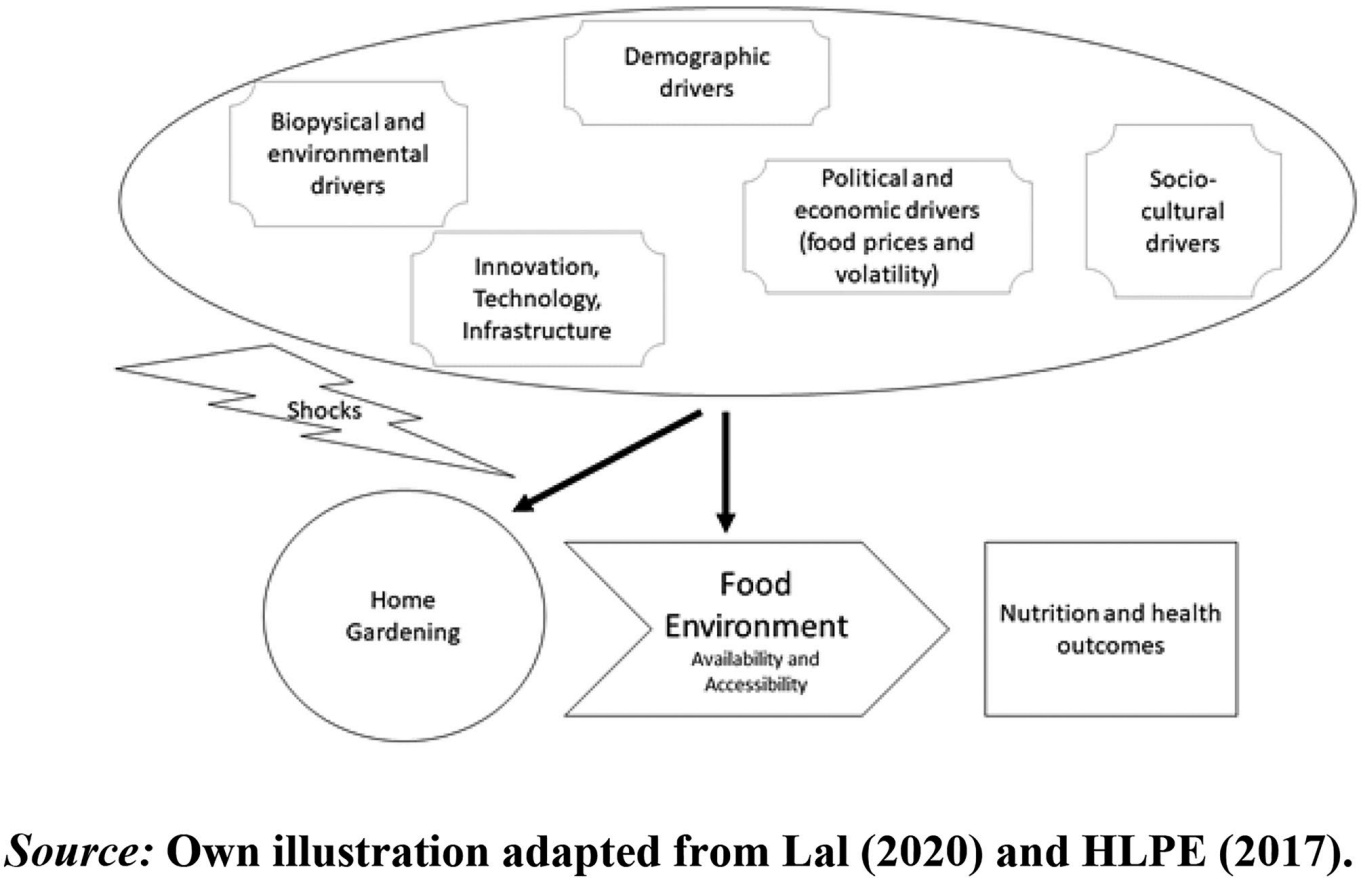
Food system framework for the relationship between home-gardening and nutrition

Figure 1 presents the conceptual relationships between home-gardening and nutrition, and health outcomes mediated by the food environment. Specifically, we aim to understand how local food systems’ characteristics affect the decision to engage in home-gardening and how that could positively affect the food and nutrition security outcomes. The potentially endogenous relationships outlined need to be addressed by the econometric approach that allows sufficient identification of the associations between home-gardening and nutrition outcomes. Besides these direct impacts on diets and nutrition, home-gardening could also positively affect environmental quality, preservation of biodiversity, and ecosystem services (Lal, 2020; Galhena et al., 2013). Notably, from a policy perspective, food system disruptions always have strong negative effects on the food environment. Therefore, in some instances, home-gardening could contribute to food system resilience by buffering market shocks to the local food environment. For instance, Galhena et al. (2013) emphasize that home-gardening is a viable option in post-conflict situations, when food systems are dysfunctional and must be rebuilt. Moreover, Seeth et al. (1998) show that home-gardens provide access to nutritious foods in the face of transformation risks when labor is not yet efficiently allocated. Meanwhile, Lal (2020) argued that the COVID-19 pandemic has created widespread disruptions in food supply chains and home gardens, particularly in urban areas. Home-gardening can overcome such short-term supply shortages.

This study focuses on home-gardens’ effects on diets, and nutrition because of the potential effects of home-gardening production diversity (Sibhatu et al., 2015; Sibhatu & Qaim, 2018; Islam et al., 2018). For instance, Baliki et al. (2019) examined an integrated home-garden intervention in Bangladesh and ascertained an increase in the supply of the following important micronutrients including iron, zinc, folate, and pro-vitamin A. Given these findings, several countries, including Ethiopia (Hirvonen & Headey, 2018), Cambodia (Schreinemachers et al., 2018), Bangladesh (Baliki et al., 2019; Schreinemachers et al., 2015), and South Africa (Bahta et al., 2018), have implemented large-scale home-garden promotion programs.

Despite the multiple benefits of home-gardens, it is neither economic nor efficient if all households, irrespective of the characteristics of the food system and the food environment, practice home-gardening to produce their own food. For instance, Castaneda-Navarrero (2021) finds that the association between the participation in home-gardening practices and food security indicators varied across the communities in the Yucatán region in Mexico. Meanwhile, Hirvonen and Headey (2018) emphasize the importance of climatic conditions, such as water availability, and the suitability of home-gardening. The household’s own small-scale food production is less beneficial if food systems function well and provide healthy food in a healthy environment at affordable prices. In such situations, rural consumers can rely on the market to obtain nutritious foods, such as vegetables and fruits (Koppmair et al., 2017; Sibhatu & Qaim, 2017). Meanwhile, if market access is limited, generally or during some part of the year, households might lack the opportunity to offer their products or labor to market and access nutritious diets (Bonuedi et al., 2021).

With few exceptions (e.g., Abdoelllah et al., 2020), the existing literature considers household theory and the determinant of smallholder commercialization only implicitly. We follow the analytical framework developed by von Braun et al. (1991) for rural Rwanda to understand the transition of households toward commercialization. Specifically, we emphasize the driving forces behind resource allocation to market versus subsistence production as important factors governing the adoption and potential socio-economic benefits of home-gardening. The model involves a distinction between subsistence farming, market production, and wage earning at resource use. It also considers “determinants, such as risk aversion, tasks, and habits that may motivate a household to maintain a certain degree of self-sufficiency even at the cost of market income forgone” (von Braun et al., 1991, p. 31). The home production, both for subsistence and the market, increases in on-farm productivity and decreases in the market wage rate. Meanwhile, the family composition and associated demand for food and non-food goods can have ambiguous effects on resource allocation between subsistence and market production. In the presence of risks, households may have specific preferences for subsistence and market production. Precisely, risk-averse households will focus on subsistence farming and accept (expected) income losses from market sales. Moreover, lower transactions and a reduction in risks associated with marketing, for instance, through cooperative membership, could reduce the preference for subsistence farming. Finally, improved access to resources, land, labor, and other inputs is expected to increase subsistence and market production, while decreasing the share of subsistence farming. This framework accounts for the endogeneity of farmers’ decision to participate in home-gardening as a means of subsistence farming. It also illustrates under which circumstances home-gardening could have greater benefits, namely, if markets are imperfect (i.e., high market risk, limited market access, and input resources).

Smallholders in Eastern and Southern Africa participate in market sales only to a limited extent; instead, food markets are dominated by larger and wealthier farmers who have access to land, livestock, capital, and improved technologies (Barrett, 2008; Carletto et al., 2017). The empirical literature provides ample evidence for the predictions of the theory of commercialization and the importance of asset ownership, resources, and transaction costs for market participation (Bellemare & Barrett, 2006). Generally, agricultural commercialization and nutrition outcomes are positively correlated (Bolarinwa et al., 2020; Carletto et al., 2017). However, studies also indicate the need to correct market imperfections to enable all farmers to participate and benefit from commercialized agriculture (Shiferaw et al., 2008; Pingali et al., 2019; Weatherspoon et al., 2021).

## Data and descriptive statistics

We use the Comprehensive Food Security and Vulnerability Analysis (CFSVA) surveys conducted by the WFP and NISR. The CFSVA is a repeated cross-sectional survey collected every three years since 2006 to measure and assess Rwanda’s current state of food and nutrition insecurity (see WFP, 2018).^1^ The regular assessment based on nationally representative data allows us to observe trends in food consumption and anthropometric status of women and children over time. It also allows us to identify demographic determinants of food insecurity and enable socio-economic analysis. For the present analysis, we use the survey waves from 2012 (sample size—10,213), 2015 (11,959), and 2018 (11,378).

A two-stage random sampling procedure was employed in all 30 Rwanda’s districts. First, 30 communities were drawn from each district. Second, 10 households per community were randomly selected. The samples of the CFSVA rounds were drawn to be nationally representative. To achieve the representativeness of the nutrition indicators at the district level, the survey captured the number of children between 5–59 months old in a household as this group accounts for about 15% of the Rwandan population (WFP, 2018). The 2012 and 2018 surveys were conducted in March and April, whereas the 2015 survey data were collected in April and May.

We use the CFSVA data sets because of their comprehensive nutrition module that includes detailed questions on dietary composition and frequency of consumption. It also includes food expenditures at the household level and food group consumption of women and children below five years old. In addition, anthropometric measures of children (e.g., height-for-age, weight-for-age, and weight-for-height) and women (e.g., body mass index [BMI]) are reported. The other questionnaire modules cover all important aspects of household demographics, livelihood activities, farm size, production diversity, market access, and consumption patterns. However, the agricultural module is not as detailed as in agricultural surveys, such as Rwanda’s Integrated Household Living Conditions Survey. For instance, only sales data but no production data are available, and inputs are measured only by dichotomous variables. However, we believe this is tolerable because we do not examine agricultural returns or yields, but we only use these indicators as control variables.

Table 1 presents detailed descriptive statistics of the variables used in the present analysis. In the whole sample, about 62% of the households (composed of about 51% and 65% of urban and rural households, respectively) participate in home-gardening. Noteworthily, the participation rate of home-gardening has slightly increased from 59% in 2012 to 64% in 2018. The mean values of the variables for households that participate and do not participate in home-gardening were provided separately. Moreover, we present the differences in means of home-gardening participants and non-participants and the respective t-value for the mean difference t-test. We utilize the standard nutrition indicators proposed in the literature (FAO, 2010). The Household Dietary Diversity Index (DDS) is measured as the number of food groups consumed by the household within the last seven days out of eight food groups: cereals and tubers, beans, vegetables, fruits, meat and fish, milk, sugar, and oil. The food consumption score (FCS) multiplies the consumption frequency over a week (maximum 7) times the nutritional value weight of the food groups and creates an aggregate. Households with a value above 35 (below 21.5) are considered having an acceptable diet (poor diet) (WFP, 2008). The child DDS score (CDDS) measures the number of food group intake (out of eight food groups) of children within the last 24 h (WHO, 2008). To measure the anthropometric status of children, we examine the height-for-age z-scores (HAZ) using the 2006 WHO Child Growth Standards (i.e., “the WHO standards”) (WHO, 2006). For women’s anthropometric status, we employ the BMI.

**Table 1:** Mean differences in variables among participants and non-participants in home-gardening

Variable	Description	Home-gardening participants N = 18,387	Non-home participants N = 11,043	Diff	T-value
*Outcome variables*					
*FCS*	Food consumption scores	48.364	46.102	2.262**	9.94
*CDDS*	Dietary diversity scores of children under 5	1.476	1.387	0.089***	4.26
*DDS*	Household dietary diversity scores	5.258	5.020	0.238***	12.77
*BMI*	Body mass index of women in reproductive age	22.744	22.801	− 0.057	− 0.88
*HAZ*	Height-for-age z-scores	− 1.668	− 1.688	0.020	0.87
*Share food exp (FES)*	Share of household expenditure on food	0.478	0.522	− 0.438***	15.4

*Control variables*					
*HH-size*	Number of household members	5.300	4.834	0.467***	17.6
*Gender-head*	Gender of household head, male = 1	0.757	0.716	0.041***	7.89
*Age-head*	Age of household head, years	47.007	45.682	1.326***	7.34
*Education*	Years of formal education	2.385	2.336	0.049**	2.85
*Land size*	Land size category	2.436	1.801	0.634***	30.61
*Rural*	Rural or urban, Rural = 1	0.146	0.233	0.086	18.94
*Access irrigation*	Access to irrigation	0.061	0.041	0.020***	7.28
*Cooperative*	Membership in a cooperative	0.243	0.162	0.081***	6.51
*Credit_access*	Access to credit	0.332	0.285	0.047	0.99
*Vegetable*	Frequency of vegetable consumption	5.353	5.049	0.811***	30.35
*Off-farm*	Engaging in off-farm activities	0.740	0.720	0.021***	3.93
*Mean_gard*	Diffusions of home gardening at the community level	0.710	0.483	0.226***	92.76
*Age_gard*	Diffusion of home gardening among the age group	0.711	0.481	0.229***	93.61
*Logtlu*	Tropical livestock unit	− 2.830	− 4.038	1.208***	31.61
*Number_crops*	Number of crops	1.736	1.258	0.477***	30.12
*Market_distance*	Distance to market (km)	2.606	2.650	− 0.044***	− 3.96
*Time_water_sourc*	Time spent to reach water source (Minutes)	1.389	1.396	− 0.007	− 0.83
*Commercialization_rate*	Mean percentage of harvest sold of top 3 crops	11.474	7.452	4.022***	20.61
*Fertilizer*	Applied chemical fertilizer to any plot = 1	0.429	0.299	0.130***	22.36
*Pesticides*	Applied chemical insecticide to any plot = 1	0.130	0.079	0.051***	13.49
*Price_index_deflated*	Deflated price index of staple crops	6.457	6.607	− 0.149***	− 15.38

*p < 0.1; **p < 0.05; ***p < 0.01

We use the standard socio-economic household and demographic controls based on extensive literature review related to food security and nutrition, home-gardening, and agricultural technology adoption (Table 1). *Off-farm* is measured as a dummy equals 1 if the household had at least one non-farm livelihood activity. The major control variables include distance to the market, time to water source, and land size, which were measured as categorical variables. Distance to the market and time to water use ranges 1–4 and 1–3, respectively, with 1 indicating the market or water source is in the village. Land size is measured by seven categories; if the household does not own agricultural land, it is denoted by 0.^2^ The commercialization rate is measured as the the share of gross value of crop sales in total gross value of production (Carletto et al., 2017).^3^ The distance to the market has improved since 2012, hence, the majority of households travel less than 60 min only. In line with this, the average commercialization rate has increased from 13.4% in 2012 to 17.3% in 2018. Meanwhile, land sizes substantially reduced over this period. We also combine household-level data with district-level real food prices for beans, cassava, maize, meat, rice, and sorghum obtained from the VAM database. The variable food price index is constructed as the average of these prices.

Home-gardening participants exhibit significantly better nutrition security outcomes and higher child nutrition outcomes than non-participating households. However, the women’s BMI was higher among non-participating versus participating households. Moreover, non-participants exhibited a higher share of food expenditures than participants. A higher household food expenditure share of total household expenditure is an indicator of low welfare status (Engel’s Law); to this extent, participants still perform better in food expenditure share. Although these comparisons give anecdotal information about the differences between households engaging in home-gardening and those not doing so, such comparisons do not consider the effect of confounding factors that could influence households’ decisions to participate in home-gardening. Therefore, we employ the ESR model that controls for both observable and unobservable factors.

Table 1A also shows home-gardening participants and non-participants have group mean differences among several observed variables. For example, gender composition of the home-gardening participants is significantly more male dominated than non-participants. Home-gardening participants are also more likely to be educated, aged, located in urban areas and near the market, engage in off-farm activities, use chemical fertilizers and pesticides, diversified and commercialized, and possess more/less livestock. Furthermore, household and landholding size and likelihood of accessing irrigation, cooperative membership is larger among home-gardening participants. Interestingly, the share of expenditure on vegetables is comparatively lower among the home-gardening participants. It is also evident that diffusions of home-gardening at the community level and among the same age group are significantly higher among the home-gardening participants, which imply the cluster or peer effects of home-gardening participation. The food price index is significantly higher among non-participants than for participants. We relate this observation to the fact that the food price index is much higher for urban households that are less likely to farm a home garden. Therefore, this difference might actually originate from cross-correlations.

## Analytical framework and empirical strategy

### Home-gardening participation

This section presents the analytical framework and the empirical strategy, describing the decisions of the households to participate in home-gardening, and how participation impacts food and nutrition security outcomes. We begin by modelling the decision to participate in home-gardening under the assumption that farmers are risk-averse and decide to either participate or not to participate in order to maximize expected net benefits (Di Falco et al., 2011). In addition, we assume that the households consist of farmers who may work in their own home-gardens rather than other plots of the households or supply labor to the market. Farmers have limitations in their ability to process new information, based on differences in human, physical, and social capital. That is, they are heterogeneous in their management abilities. In particular, farmers may differ in land availability and land productivity/suitability, financial resources, family size and age of the household head, participation in social gathering, and short-/long-term home-gardening participation. Under this assumption, let the expected net benefits household *i* derived from the participation in home-gardening be denoted by ${H}_{i}^{*}$. The expected net benefits cannot be observed but can be expressed as a function of observed characteristics represented by a vector ${{\varvec{X}}}_{i}$. We specify the home-gardening participation decision problem in a latent variable model as

1 $${H}_{i}^{*}={\alpha X}_{i}+ {\varepsilon }_{i},\ H_i=1\ if\ [{H}_{i}^{*}>0],\ {H}_{i}=0,\mathrm{ otherwise}$$

where ${H}_{i}$ is an observable binary variable representing the decision to participate (${H}_{i}=1$) or not to participate in (${H}_{i}=0$) home-gardening; $\alpha$ is a vector of parameters to be estimated. ${{\varvec{X}}}_{i}$ is a vector of farm-level and household characteristics, access to land, household expenditure share on food, asset holdings, and engagement in off-farm activities. The error term ${\varepsilon }_{i}$ *is* assumed to be normally distributed and uncorrelated with ${{\varvec{X}}}_{i}$. The probability that a farmer participates home-gardening can be expressed as

2 $$Pr({H}_{i}=1) = Pr({H}_{i}^{*}>0) =pr\ ({\varepsilon }_{i}>-{\alpha X}_{i} =1-F(-{\alpha X}_{i})$$

### Impact of home-gardening

Given that this study examines the drivers of participating in home-gardening and the impact of participation on food and nutrition security, we represent the outcome equation as

3 $${Q}_{i}=\gamma {\mathrm{\rm K}}_{i}+\delta {H}_{i}+{\mu }_{i}$$

where ${{\varvec{Q}}}_{i}$ refers to the vector of food and nutrition security outcomes captured here as DDS, CDDS, FCS, HAZ, BMI, and household expenditure share on food (FES). ${H}_{i}$ is as defined earlier, and ${\mathbf{K}}_{i}$ is a vector of explanatory variables that include household-level characteristics and location (district) fixed effects. Moreover, $\gamma$ is a vector of parameters to be estimated, whereas ${\mu }_{i}$ is the error term assumed to be uncorrelated with both ${\mathbf{K}}_{{\varvec{i}}}$ and ${H}_{i}$. The parameter $\delta$ captures the impact of participation on household food and nutrition security outcomes. However, estimating the impact of home-gardening using Eq. 3 could result in biased estimates, because it assumes that farmers’ decision to participate is exogenous, but it is endogenous because the decision to participate is self-determined (Heckman, 1979). Households that decide to participate in home-gardening may also be systematically different from those that do not participate. In addition, unobservable factors influencing the participation decision may also affect the food and nutrition security outcomes, resulting in inconsistent estimates of the parameters $\gamma$ and $\delta$.

To account for the selectivity bias due to observable and unobservable factors, we employ a simultaneous equation model with endogenous switching, where the food and nutrition security outcomes of households (i.e., DDS, FCS, WDDS, HAZ, and FES) are specified in two regimes:

4 $$\mathrm{Regime\ 1}:\ {Q}_{i1}={\gamma }_{1}{\mathrm{K}}_{i}+{\mu }_{i1}\qquad{if}\ \qquad{H_i=1}$$

5 $$\mathrm{Regime\ 2:}\ {Q}_{i0}={\gamma }_{0}{\mathrm{K}}_{i}+{\mu }_{i0}\qquad{if}\qquad{H_i=0}$$

where ${{\varvec{Q}}}_{i1}$ and ${{\varvec{Q}}}_{{\varvec{i}}0}$ are the food and nutrition security outcome variables for participants and non-participants, respectively. Meanwhile, ${\mathbf{K}}_{i}$ is a vector of the aforementioned farm and household-level characteristics.

Because farmers self-select into participant and non-participant categories, the covariances between the error terms of the participation decision (Eq. 1) and the outcome (Eqs. 4 and 5) may be non-zero (i.e., corr(${\mu }_{ij}{\varepsilon }_{i})=\rho$). We subsequently estimate selectivity^4^ corrected model of food and nutrition security outcomes, given the home-gardening participation:

6 $$E({Q}_{i1}|{H}_{i}=1)={\gamma }_{1i}{\mathrm{K}}_{i}+{\sigma }_{1\varepsilon }{\lambda }_{1i}$$

The expected ${\varvec{Q}}$ value of farmer $i$ if he had chosen not to adopt (counterfactual case) is

7 $${E(Q}_{i0}\left|{H}_{i}=1\right)={\gamma }_{0i}{\mathrm{K}}_{i}+{\sigma }_{0\varepsilon }{\lambda }_{1i}$$

where ${\lambda }_{1i}$ and ${\lambda }_{0i}$ refer to the inverse Mills’ ratios (selectivity terms), and ${Q}_{i}$ and ${H}_{i}$ are as defined already. Meanwhile, ${\sigma }_{1\varepsilon }$ and ${\sigma }_{0\varepsilon }$ represent the covariances of the error term ${{\varvec{\varepsilon}}}_{i}$ in the participation equation and that of ${u}_{1i}$ and ${u}_{0i}$ in Eqs. (4) and (5), respectively. A change in the outcome due to participation, which is termed the average treatment effect on the treated (ATT), is expressed as the difference in the expected outcomes between Eqs. (6) and (7) (Lokshin & Sajaia, 2004):

8 $$\mathrm{ATT}=E({Q}_{1i}| {H}_{i}=1)-E({Q}_{0i}\left| {H}_{i}=1\right)=({\gamma }_{1i}-{\gamma }_{0i}){K}_{i}+{\lambda }_{1i}({{\sigma }_{1}}_{\varepsilon }-{{\sigma }_{0}}_{\varepsilon })$$

An additional concern in estimating the participation equation is the potential endogeneity of the off-farm work participation variable. This is because income earned from off-farm work can be invested in technologies or yield enhancing inputs that make home-gardening more attractive. However, engaging in off-farm work may lead to reduced time allocation to on-farm practices (labor-loss effect). In addressing this potential endogeneity, we use the control function (CF) approach outlined by Wooldridge (2015) to address this problem. The approach involves the specification of the potential endogenous variable (i.e., off-farm work participation) as a function of other explanatory variables in the home-gardening participation equation (Eq. 1), with a set of instrument(s) in a first-stage regression. In the second stage, the observed values of off-farm work participation variable and the residuals from the first-stage probit regression are included as covariates in Eq. (112571). The inclusion of the residuals serves as a CF, enabling consistent estimation of the potentially endogenous variable and serving as a robust test of exogeneity of the off-farm work participation variable (Wooldridge, 2015).

In estimating the ESR count data model, finding a set of suitable exogenous variables during the simultaneous estimation of the treatment (home-gardening participation) and outcome equations is necessary in identifying Eqs. (4) and (5). A suitable identification strategy is to employ a variable that significantly influences the decision to participate but does not directly influence food and nutrition security outcomes. The variables we used as instruments are cooperative membership, the diffusion of home-gardening at the community level, and the diffusion of home-gardening among the age group of the household heads at the district level. Cooperative membership has been observed to affect farmers’ participation decisions directly (e.g., Abebaw & Haile, 2013; Ma & Abdulai, 2016). Furthermore, the diffusion of home-gardening among age cohorts at the household level might influence the spread of participation among households. We conducted a placebo regression to confirm the admissibility of these instruments.

Although cooperative membership appears to meet the exclusion restriction in the model, some real-life situations may affect its suitability as an instrument. For instance, one might argue that, in some real situations, the variable could influence the farm household probability of increasing production and thus food and nutrition security. In addition, farmers who have access to nutrition information through cooperative membership or age cohorts may adjust input levels and techniques throughout the season to deal with the weather, which would affect farm revenue or dietary choices. These possibilities were however, absent in our case, as confirmed by the falsification tests of these instruments (See Table 9 in appendix). The instruments were sufficient for identification and were therefore excluded. In the CF approach, we used distance to market as an instrument in the first-stage regression of off-farm work participation. This variable was therefore excluded from the selection and outcome equations during the estimation.

## Results and discussion

### Drivers of home-gardening participation

Table 2 presents the drivers of a household’s decision to participate in home gardening. These results are obtained from the probit component (first-stage) of the ESR model. The Wald statistics indicate an acceptable model fit. Moreover, the model’s potential endogeneity of off-farm work participation is confirmed as indicated by the moderately significant (at 10% margin of error) residual (*Off_farmresid*).^5^

**Table 2:** Drivers of home-gardening participation

Variable	Coeff	Std Err.	Z-value
*hh_size*	0.026***	0.009	2.770
*age_head*	0.001	0.001	1.100
*education*	0.065***	0.023	2.800
*access_irrigation*	0.016	0.082	0.200
*landcat2*	0.077	0.050	1.550
*landcat3*	0.055	0.051	1.080
*landcat4*	0.081	0.064	1.270
*landcat5*	0.237***	0.050	4.700
*landcat6*	0.262***	0.091	2.880
*landcat7*	0.242***	0.070	3.450
*marketcat1*	− 0.045	0.048	− 0.940
*marketcat2*	0.015	0.022	0.700
*marketcat3*	− 0.041	0.026	− 1.570
*watersource1*	− 0.016	0.034	− 0.480
*watersource3*	0.045	0.080	0.560
*Off-farm activities*	0.190	0.126	1.500
*log_TLU*	0.026***	0.006	4.260
*number_crops*	0.025	0.016	1.510
*commercialization_rate*	0.001	0.001	1.160
*fertilizer*	0.138***	0.040	3.450
*pesticides*	− 0.033	0.070	− 0.470
*price_index_deflated*	0.081***	0.019	4.210
*Off_farmresid*	− 0.072*	0.043	− 1.680
*gender_head*	− 0.085*	0.048	− 1.780
*age_gard*	2.721***	0.089	30.680
*mean_gard*	2.672***	0.092	29.200
*cooperative*	0.189***	0.023	8.280
No. Observations	22670		

***Diagnostics***			
Wald Chi	3397.79		
Prob > χ^2^	0.000		
Log Pseudolikelihood	− 23813254		

Cluster Robust standard errors in parentheses *p < 0.1; ***p < 0.01

From the results, household socio-economic variables, such as household size and the head’s level of education, were positively and significantly associated with the household’s decision to participate in home-gardening. In addition, female household heads were significantly more likely to participate in home-gardening than male household heads. The significant correlation between household size and home-gardening participation is also consistent with the findings of Mufeeth et al. (2021), who observed family labor as a critical source of labor supply for cultivating home gardens. In addition, Gbedomon et al. (2015) observe a strong correlation between education and the gender of the household head and the probability of owning a home garden in Benin. Educated farmers may have better access to information and a more profound understanding of the nutritional and other benefits of home gardening; they can also transform new information more quickly and effectively (Foster & Rosenzweig, 2010). Galhena et al. (2013) find that the gender dimension of home-gardening is culture-specific and varies across countries. In line with several other studies (e.g., Kabir & Webb, 2009; Gbedomon et al., 2017; Rybak et al., 2018), we observe that female-headed households are more likely to participate in home-gardening in Rwanda. Specifically, female-headed households are 8.5% less likely to cultivate home gardens. The effect is relatively moderate, which may be due to the equal split of vegetable farming between male and female members of the household in Rwanda. This result is also in line with the adoption of nutrition-sensitive agriculture, which claims that women are the drivers of nutrition-sensitive agriculture change (Rukmani et al., 2019). Among the input variables, only access to fertilizers was positively and significantly associated with home-gardening participation. Interestingly, livestock ownership, expressed in tropical livestock units (TLU) (a proxy for household wealth), positively and significantly influenced household decisions to practice home gardening, a finding that is consistent with Bahta et al. (2018). This association could be related to wealth represented by livestock ownership, but complementarity also exists between livestock rearing and home-gardening, as manure is often used to fertilize home-gardens.

We also ascertain a significant correlation between land ownership and home-gardening participation. Specifically, households in all land ownership categories are more likely to participate in home-gardening than landless households with households who own more than 0.5 ha of land, showing more likelihood. The distance to market was not significantly related to home-gardening participation, which corroborates the finding of Bahta et al. (2018) but not that of Hirvonen and Headey (2018), who observe that better market access encouraged home-gardening participation in Ethiopia. Similarly, we do not find a significant relationship between the commercialization rate and home gardening participation. The absence of a negative correlation between commercialization and home-gardening emphasizes that subsistence farming and commercialization are not contradictory. Our result on the effect of commercialization is different from that of Abdoellah et al. (2020), who observed a high level of commercialization of home-gardens in Indonesia. However, in our sample, 98% of the households reported using the home-garden produce for home consumption. We also find that households that had an off-farm livelihood activity are more likely, although not statistically significant, to participate in home-gardening. The coefficient of the variable number of crops is positive and statistically significant, implying that crop diversification also drives home-gardening participation, all things being equal. This finding is consistent with that of Gbedomon et al. (2017), who noted that production diversity is among the important functions of home-gardening in Africa.

Furthermore, we find a positive correlation between home-gardening participation and the food price index. This implies that higher market prices for staple crops could also influence farmers’ decisions to minimize market risks and use available land around the household to produce nutritious foods. Last but not the least, most of our findings on drivers of home-gardening participation (e.g., family size, education, farm size, access to market, and livestock ownership) also corroborate with the broader agricultural technology adoption determinants (Feder et al., 1985; Feder and Umali, 1993). In summary, we find evidence for the hypothesis that home-gardening fulfills a risk management strategy, particularly for households with limited access to land.

The results also show that all instruments are statistically significant in the home-gardening participation model, suggesting the relevance of these instruments. Specifically, cooperative membership positively and significantly influences households’ decisions to participate in home-gardening, implying that belonging to a cooperative, especially farmers’ cooperative, could serve as a major source of information that could influence household decisions about home-gardening, as shown by several other studies (e.g., Di Falco et al., 2011; Ma & Abdulai, 2016). Furthermore, diffusion of home-gardening among cohorts at the village level (*mean_garden*) and among age groups (*mean_age*) significantly drives home-gardening participation. Intuitively, these instruments are less likely to influence home-gardening participation through the extent of diffusion directly and thus farmers’ knowledge about the benefits of home-gardening among the households. We believe that these variables do not directly affect household or child nutrition outcomes. Therefore, the instruments were excluded from the outcome equations in the ESR model. Specifically, the variable cooperative positively and significantly influences household decision to own a home-garden, implying that belonging to a cooperative, especially farmers’ cooperative group could serve as a major source of information that could influence households decisions about home-gardening. Furthermore, diffusion of home-gardening among cohorts at the village level (*mean_garden*) and among age groups (*mean_age*) significantly drive home-gardening participation. Intuitively, this instrument likely influences participation in home-gardening directly, but not household or child nutrition outcomes.

### Impact of home-gardening on food security and child nutrition

This section discusses the results of the impacts of home-gardening on food and nutrition security. This is obtained by the treatment effects on the treated (ATT), measured as the mean difference between the expected outcome of participants of home-gardening and what they would have obtained if they had not participated. We focus on discussing these ATTs in the interest of brevity. The results are presented in Tables 3–6. However, the drivers of the food and nutrition security outcomes are presented in Tables 7 and 8 in the appendix, but are not discussed. The results reveal significant correlation coefficients (*rho*) associated with CDDS, WBMI, and FES for the participant category, which indicates the presence of selectivity bias, and justifies the use of the ESR approach in this study.

**Table 3:** ATT of home-gardening participation: pooled data and by location

Sample	Food and nutrition Security indicator	Mean outcome of HG participants	Mean outcome of counterfactual (Non-participants)	ATT/Std. Err.
Pooled	FCS	48.068 (0.075)	45.974(0.073)	2.094***(0.018)
Urban		55.461(0.289)	53.191(0.293)	2.270***(0.047)
Rural		47.157(0.072)	45.084(0.071)	2.073***(0.019)
Pooled	DDS	5.232(0.006)	4.963(0.006)	0.269***(0.006)
Urban		5.762(0.021)	5.531(0.023)	0.231***(0.005)
Rural		5.167(0.006)	4.893(0.006)	0.274***(0.02)
Pooled	CDDS	1.869(0.005)	1.657(0.004)	0.211***(0.002)
Urban		1.697(0.014)	1.562(0.011)	0.134***(0.006)
Rural		1.889(0.005)	1.669(0.004)	0.221***(0.002)
Pooled	WBMI	22.608(0.007)	22.326(0.007)	0.282***(0.005)
Urban		23.202(0.030)	22.768(0.027)	0.433***(0.018)
Rural		22.535(0.006)	22.272(0.007)	0.263***(0.006)
Pooled	HAZ	− 1.678(0.003)	− 1.746(0.003)	0.069***(0.002)
Urban		− 1.452(0.012)	− 1.600(0.010)	0.148***(0.007)
Rural		− 1.705(0.005)	− 1.764(0.003)	0.059***(0.002)
Pooled	FES	0.452(0.001)	0.465(0.001)	− 0.013***(0.0003)
Urban		0.454(0.002)	0.463(0.003)	− 0.009***(0.001)
Rural		0.452(0.001)	0.466(0.001)	− 0.014***(0.0003)

***p < 0.01

Table 3 presents the impact (ATTs) of home-gardening on DDS, CDDS, FCS, women’s BMI (WBMI), HAZ, and FES. As shown, participation in home-gardening exerts a positive and significant impact on all the food and nutrition security outcome variables at least at the 1% level except FES. In particular, participation in home-gardening significantly contributes about 4.6% and 5.4% increase in FCS and DDS, respectively, compared to non-participation. The gain in CDDS associated with participation is even much higher (12.7%), relative to non-participation. The results also reveal similar effects for the anthropometric outcomes: WBMI and HAZ. We find that children living in households that cultivate home gardens gain about 0.07 standard deviations in height for their age, whereas WBMI was averagely 0.28 points higher than households without home gardens. Lastly, in line with our expectations, participation in home-gardening significantly reduces FES by about 2.8% as compared to non-participation.

These findings are consistent with the general view that home-gardening is positively associated with improved dietary diversity reported in previous nutrition-sensitive studies (e.g., Ruel & Alderman, 2013), and home-gardening studies in Mexico (e.g., Castañeda-Navarrete, 2021), in South Africa (e.g., Bahta et al., 2018), in Maynmar (e.g., Rammohan et al., 2019), and in Bangladesh (e.g., Bushamuka et al., 2005; Schreinemachers et al., 2016). Moreover, our findings imply that home-gardening holds great potential for improving household and child nutrition outcomes, such as WBMI and HAZ. However, these findings are contrary to that of Depenbusch et al. (2021), who observed no statistically significant effect of home-gardening on diets in Kenya, Uganda, and Tanzania.

Conversely, results also show a decrease in the share of household food expenditure (FES) by up to 2.8% among participants in Rwanda, which is in line the theory that an increase in household income (expenditure) decreases the percentage share on food, all other factors remaining constant (Grigg, 1994). This is also consistent with the findings of Schupp and Sharp (2012) and Brown and Kulcsar (2001), who observe that participation in home-gardening may be largely motivated by economic necessity and an adaptive strategy, particularly among low-income households.

### Heterogeneous impacts of home-gardening on food and nutrition security

As aforementioned, the estimation of ATT assumes common treatment effects. However, treatment effects can vary in terms of socio-economic groups, including, income, poverty, geographical location, and access to resources, within the same treatment group (Ali and Abdulai, 2010; Tambo et al., 2021). Here, we identify whether the treatment effect on food and nutrition security, varies by location (rural/urban), land size/plot ownership, the extent of commercialization (proportion of the harvest sold), and price levels. In Table 2, we show that land/plot ownership, the extent of commercialization, and price levels have differential effects on home-gardening participation. Therefore, we disaggregated the ATT based on these variables to determine whether all these categories of participants achieve similar or differential benefits from home-gardening. The results are presented and discussed in the following subsections.

#### Heterogeneous impact of home-gardening by location (Rural/Urban)

Table 3 shows the ATT disaggregated by rural–urban dichotomy. The results show that home-gardening participation is significantly associated with improved food and nutrition security outcomes for all households regardless of their location. However, the magnitudes of the ATTs generally point to a greater impact of home-gardening participation among rural households, suggesting a higher likelihood of home-gardening participants in rural areas benefiting more from improved household and child dietary quality and FCS. Specifically, although participation has resulted in 5% increase in FCS among the ATT in rural households, the increase in FCS among urban households is about 3%. In the case of DDS, the change is 6.6% for rural households, whereas that of urban households is about 3.8%. Conversely, child HAZ score and women BMI are better for home-gardening participants in urban areas than those in rural areas. Furthermore, the FES associated with participating households in rural areas declined by 3.5%, whereas those in urban households increased marginally (< 1%). These findings are consistent with that of Bahta et al. (2018) in rural South Africa where home-gardening led to about 45% decline in food insecurity.

#### Heterogeneous impacts of home-gardening by land sizes/land ownership

Table 4 presents the results of the impacts of home-gardening on food and nutrition security based on land sizes. This is necessary because land ownership is assumed to be a key determinant of households’ decision to participate in home-gardening as indicated by its positive and significant effect on the probability of participating in home-gardening (Table 2). Interestingly, concerning DDS and FCS, irrespective of landholding categories, all households benefited from the participation in home-gardening.

**Table 4:** Impacts of home-gardening participation on food and nutrition security by land sizes

Land size (ha)	Food and nutrition Security indicator	Mean outcome of HG participants	Mean outcome of counterfactual (Non-participants)	ATT/Std. Err.
No land		53.438 (0.267)	51.488 (0.268)	1.950***(0.045)
0–0.1	FCS	41.553(0.145)	39.353(0.140)	2.199***(0.042)
0.1–0.19		44.524(0.139)	42.856(0.135)	1.668***(0.042)
0.2–0.49		46.222(0.121)	44.879(0.118)	1.342***(0.037)
0.5–0.99		49.799(0.134)	46.563(0.132)	3.236***(0.038)
1.0–1.99		54.051(0.179)	51.321(0.176)	2.729***(0.049)
2–5		62.587(0.364)	61.668(0.361)	0.919***(0.083)

No Land		5.637***(0.020)	5.409***(0.021)	0.229***(0.004)
0–0.1	DDS	4.800(0.013)	4.439(0.013)	0.359***(0.04)
0.1–0.19		4.985(0.012)	4.742(0.013)	0.243***(0.013)
0.2–0.49 a		5.064(0.011)	4.880(0.011)	0.184***(0.003)
0.5–0.99		5.363(0.011)	5.028(0.012)	0.335***(0.003)
1.0–1.99		5.716(0.015)	5.384(0.016)	0.332***(0.005)
2–5		6.036(0.029)	5.959(0.031)	0.077***(0.008)

No land		− 1.489 (0.011)	− 1.68 (0.009)	0.197***(0.006)
0–0.1	HAZ	− 1.742(0.006)	− 1.936(0.006)	0.194***(0.004)
0.1–0.19		− 1.794(0.006)	− 1.868(0.006)	0.074***(0.004)
0.2–0.49		− 1.797(0.005)	− 1.773(0.005)	− 0.024***(0.004)
0.5–0.99		− 1.648(0.006)	− 1.759(0.005)	0.11***(0.004)
1.0–1.99		− 1.578(0.008)	− 1.455(0.007)	− 0.123***(0.005)
2–5		− 1.124(0.013)	− 1.184(0.013)	0.059***(0.009)

No Land		23.510(0.019)	23.409(0.025)	0.101***(0.016)
0–0.1	WBMI	22.527(0.015)	22.032(0.014)	0.495***(0.011)
0.1–0.19		22.585(0.014)	21.945(0.013)	0.640***(0.011)
0.2–0.49		22.451(0.013)	21.892(0.012)	0.559***(0.009)
0.5–0.99		22.426(0.014)	22.249(0.013)	0.177***(0.011)
1.0–1.99		22.410(0.018)	22.879(0.017)	− 0.469***(0.014)
2–5		23.258(0.034)	23.175(0.028)	0.084***(0.024)

No Land		0.493(0.002)	0.501(0.002)	− 0.008***(0.001)
0–0.1	FES	0.512 (0.002)	0.550 (0.001)	− 0.037***(0.003)
0.1–0.19		0.493(0.001)	0.500(0.001)	− 0.037***(0.001)
0.2–0.49		0.460(0.001)	0.490(0.001)	− 0.030***(0.006)
0.5–0.99		0.413(0.001)	0.415(0.001)	− 0.002***(0.001)
1.0–1.99		0.359(0.002)	0.373(0.002)	− 0.014***(0.001)
2–5		0.276(0.003)	0.301(0.003)	− 0.025***(0.001)

***p < 0.01

Even participating households that do not own land significantly obtained improved nutrition compared to non-participants. This observation is important and signifies the essential role of home-gardening in minimizing food and nutrition insecurity even among the landless. Similarly, results reported from Maynmar indicated significant effect of home-gardening on improve food security and dietary diversity among the rural landless households (e.g., Rammohan et al., 2019). Furthermore, the impact of home-gardening on child HAZ score varies over land size, with the landless and near landless (0–0.1 ha) households experiencing higher HAZ scores. These results imply that home-gardening is a potential intervention for reducing child stunting among the resource poor and vulnerable households in developing countries. WBMI shows an inverted U-shaped relationship with landholding among the home-gardening participants. Food expenditure share (FES) shows a significant decrease among participants of different land holding categories. This implies that home-gardening can be used to achieve enhanced welfare through a decrease in household consumption expenditure share on food.

#### Home gardening, food and nutrition security, and extent of commercialization

Table 5 presents the results on the impact of home-gardening disaggregated by the extent/level of commercialization (i.e., 0%, 1–25%, 25–50%, and more than 50% level of commercialization) on food and nutrition security.

**Table 5:** Impact of home-gardening on food and nutrition security by extent of commercialization

Sample	Food and nutrition Security indicator	Mean outcome of HG participants	Mean outcome of counterfactual(Non-participants)	ATT/Std. Err.
Percentage of sales (%)	FCS	45.515 (0.127)	43.401(0.127)	2.114***(0.028)
1–25		47.356 (0.106)	45.133(0.104)	2.222***(0.029)
25–50		51.597(0.135)	49.128(0.133)	2.470***(0.034)
Above 50		55.958(0.265)	53.26483(0.256)	2.693***(0.067)
	DDS	5.022(0.010)	4.760(0.011)	0.262***(0.003)
1–25		5.025(0.015)	4.759(0.016)	0.265***(0.005)
25–50		5.369(0.008)	5.098(0.009)	0.271***(0.003)
Above 50		5.903(0.021)	5.591(0.023)	0.312***(0.007)
	HAZ	− 1.753(0.005)	− 1.857(0.004)	0.104***(0.003)
1–25		− 1.775(0.007)	− 1.834(0.007)	0.059***(0.005)
25–50		− 1.630(0.004)	− 1.674(0.004)	0.044***(0.003)
Above 50		− 1.384(0.011)	− 1.433(0.011)	0.049***(0.008)
	WBMI	22.690(0.012)	22.317(0.013)	0.373***(0.008)
1–25		22.371(0.017)	22.088(0.018)	0.283***(0.015)
25–50		22.6310(0.013)	22.369(0.015)	0.262***(0.114)
Above 50		22.985(0.026)	22.701(0.029)	0.284***(0.020)
	FES	0.505(0.001)	0.524(0.001)	− 0.019***(0.000)
1–25		0.451(0.001)	0.466 (0.001)	− 0.014***(0.000)
25–50		0.396 (0.001)	0.403(0.001)	− 0.007*** (0.001)
Above 50		0.334 (0.003)	0.338*** (0.003)	− 0.004*** (0.001)

***p < 0.01

The results show that although participation impacts on most food and nutrition security indicators (DDS, CDDS, and FCS) consistently increase with increasing level of commercialization, the effect of home-gardening participation on the FES decreased. This finding is consistent with a priori expectation, suggesting that participation in home-gardening combined with an effective level of commercialization (e.g., efficient markets) could enhance various dimensions of household and child nutrition. This result is consistent with agricultural commercialization literature that emphasizes enhancing market access to make smallholders more nutrition-sensitive (Carletto et al., 2017; Ogutu et al., 2020; von Braun & Kennedy, 1994). Therefore, rather than substituting commercialization, home-gardening impacts on food and nutrition security increases with increasing level of commercialization, confirming the complementarity hypothesis.

#### Home gardening, food and nutrition security, and proximity to markets

The results in Table 6 show the impact of home-gardening on food and nutrition security and its nexus with market proximity (minutes in walking to the nearest market). At different market proximity levels (farmgate/village level) to distant markets, it takes > 120 min to the market. Specifically, although participants’ FCS and DDS increased in different markets (nearby or distant markets), their FES decreased, suggesting the potential of home-gardening to enhance market participation and improve food and nutrition security. Therefore, in developing countries, where food access can be a problem due to poor market access, home-gardening can supplement food supply and hence, improve food and nutrition security. This finding is consistent with that of Abdoellah et al. (2020), who indicated that during periods of income shortfall, home-gardens that produce for markets could provide a stable source of fresh and nutritious food for households in Rwanda.

**Table 6:** Impact of home-gardening on food and nutrition security by market distances

Sample	Food and nutrition security indicator	Mean outcome of HG participants	Mean outcome of counterfactual (Non-participants)	ATT/Std. Err.
Village	FCS	52.114 (0.245)	51.818 (0.2389)	0.054***(0.297)
Less than 60 min		49.100 (0.117)	46.805 (0.115)	2.294***(0.027)
60–120 min		47.032(0.136)	44.79122	2.240***(0.032)
> 120 min		45.66914 (0.149)	43.388 (0.143)	2.281***(0.037)
Village	DDS	5.500 (0.0192)	5.442 (0.020)	0.058***(0.005)
Less than 60 min		5.328 (0.009)	5.045 (0.009)	0.283***(0.002)
60–120 min		5.159 (0.0109)	4.842 (0.011)	0.317***(0.002)
> 120 min		5.025 0(.012)	4.754 (0.012)	0.270***(0.003)
Village	HAZ	− 1.403 (0.009)	− 1.649 (0.009)	0.246***(0.005)
Less than 60 min		− 1.64 (0.004)	− 1.7261 (0.004)	0.079***(0.003)
60–120 min		− 1.722 (0.005)	− 1.686 (0.005)	− 0.035***(0.003)
> 120 min		− 1.796 (0.006)	− 1.907 (0.006)	0.111***(0.004)
Village	WBMI	22.811 (0.023)	22.330 (0.024)	0.481***(0.017)
Less than 60 min		22.795 (0.010)	22.379 (0.011)	0.416***(0.008)
60–120 min		22.426 (0.012)	22.323 (0.013)	0.103***(0.009)
> 120 min		22.392 (0.014)	22.224 (0.014)	0.168***(0.011)
Village		0.408 (0.003)	0.421(0.002)	− 0.013*** (0.001)
Less than 60 min	FES	0.454 (0.001)	0.4725 (0.001)	− 0.019*** (0.000)
60–120 min		0.452 (0.001)	0.460 (0.001)	− 0.008*** (0.001)
> 120 min		0.462 (0.002)	0.483 (0.003)	− 0.021*** (0.001)

Robust standard errors in parentheses ***p < 0.01

## Conclusions and policy implications

The level of food and nutrition insecurity in sub-Saharan Africa is a major concern, and remains an important development challenge for African policymakers and the international development community. In this study, we provide results on the impact of home-gardening on food and nutrition security in different market settings and for different household types. We used Rwanda as a case study to understand how home-gardening could influence food and nutrition security. We employ an ESR model to a nationally representative dataset with diverse food and nutrition security information from Rwanda. The results show that households that cultivate home-gardens have more diverse diets and better nutritional outcomes than those who do not cultivate home-gardens. These benefits are greater even if households have limited access to land and live further away from markets. By contrast, we ascertain that the benefits of home-gardening are positive irrespective of the level of participation in commercial agriculture. This implies that all categories of commercial farmers could successfully practice home-gardening as a risk management tool against market imperfections. Meanwhile, the results revealed that variables such as education, farm size, access to irrigation, livestock ownership, agricultural diversification, cooperative membership, and price positively and significantly influence the participation in home-gardening in Rwanda. The policy implication is that enhancing access to small-scale irrigation and facilitating access to markets and education are essential to the establishment of home-gardens in Rwanda.

From a food system’s perspective, both urban and rural participants in home-gardens could achieve food and nutrition security. This implies that promotion of domestic food sovereignty and sustainable system in the context of limited land could be achieved through home-gardening. In addition, this study contributes to the wider literature on the agricultural commercialization of smallholder farmers in sub-Saharan Africa. Specifically, we observe that home-gardening and agricultural commercialization could be complementary even with limited market access. Last, we add to the policy debate in Rwanda that has focused on promoting agricultural commercialization through its agricultural policies, particularly the Crop Intensification Program,^6^ in showing that subsistence farming alongside smallholder commercialization can achieve progress in reducing malnutrition in the country.

The findings have important policy implications for food and nutrition security in developing countries. First, policymakers need to pay more attention to home-gardening as it enhances the availability of nutritious foods, particularly where markets are imperfect. Second, existing agricultural programs must consider smallholders’ desire to maintain a certain level of subsistence farming such as home-gardening as a strategy to ensure food sovereignty and cultural independence regarding indigenous foods. For instance, input subsidy and distribution programs could be expanded to include vegetable crops and agricultural extension services, including agricultural training for home-gardening food production. Furthermore, in the medium to long term, supporting homestead vegetable production could create incentives among farmers to engage in commercial production that offers great potential to the agricultural sector in Rwanda. The positive impact of home-gardening on nutritional outcomes supports Masset et al.’s (2012) argument that households that grow their own food enjoy better food security and nutrition security. The positive impact of home gardening on food and nutrition security calls for both participating and non-participating households in Rwanda to embrace home gardening as a way of augmenting their food and nutrition security needs. This study is limited in revealing information on how smallholder farmers’ resource allocation between subsistence and market production, as well as the role of subsistence farming in risk management in the presence of market imperfections. In this context, home-gardening interventions in different contexts and in combination with agricultural commercialization programs could be rolled out to understand the interactions and to utilize synergies and complementarities of subsistence farming and commercialization.

### Electronic supplementary material

Below is the link to the electronic supplementary material.

Supplementary file1 (DOCX 27 KB)

Supplementary file2 (DOCX 133 KB)

## Appendix

**Table 7:** Drivers of food and nutrition security outcomes (DDS, CDDS, and FCS)

	FCS				DDS				CDDS			
Variable	participants		Non- participants		participants		Non- participants		participants		Non- participants	
	Coef.	Robust Std. Err.	Coef.	Robust Std. Err.	Coef.	Robust Std. Err.	Coef.	Robust Std. Err.	Coef.	Robust Std. Err.	Coef.	Robust Std. Err.
*HH-size*	0.274	0.273	0.251*	0.147	0.011	0.021	0.018***	0.005	0.010	0.011	0.016***	0.005
*Gender-head*	− 0.005	0.285	− 0.032	0.284	− 0.039	0.045	− 0.054	0.048	− 0.092**	0.037	− 0.060***	0.023
*Age-head*	0.005	0.016	− 0.036	0.024	− 0.007***	0.001	− 0.012***	0.003	− 0.007***	0.001	− 0.006***	0.002
*Education*	3.292***	0.771	3.202***	1.155	0.264***	0.034	0.254***	0.070	0.038***	0.008	0.034	0.030
*Irrigation*	1.618**	0.828	− 0.833	1.167	0.165***	0.050	− 0.041	0.062	0.098*	0.055	0.055	0.153
*Landcat2*	− 5.693***	2.121	− 5.973***	1.877	− 0.385**	0.176	− 0.465***	0.108	− 0.156***	0.049	0.116***	0.045
*Landcat3*	− 3.127*	1.904	− 2.855	1.877	− 0.229	0.168	− 0.197*	0.108	− 0.059	0.072	0.032	0.032
*Landcat4*	− 2.377	1.778	− 1.621	1.663	− 0.204	0.165	− 0.102	0.112	− 0.062	0.064	0.026	0.034
*Landcat5*	− 0.757	1.870	− 1.503	1.640	− 0.071	0.166	− 0.110	0.103	− 0.174**	0.083	− 0.015	0.028
*Landcat6*	1.473	1.715	1.365	1.397	0.135	0.157	0.107	0.115	− 0.123	0.092	− 0.005	0.070
*Landcat7*	5.658***	1.634	7.507***	2.959	0.162	0.162	0.375	0.239	− 0.034	0.106	0.026	0.072
*Marketcat1*	2.420	1.731	4.679**	2.253	0.183	0.125	0.440***	0.140	0.091	0.103	0.086**	0.039
*Marketcat2*	1.786**	0.849	2.114**	0.984	0.186**	0.090	0.216***	0.071	0.055*	0.030	0.047	0.052
*Marketcat3*	− 0.100	0.321	0.148	0.488	0.024	0.049	− 0.001	0.081	− 0.035	0.036	− 0.006	0.040
*Watersource1*	1.286**	0.654	1.239**	0.617	0.098	0.064	0.139**	0.072	0.040	0.073	0.007	0.017
*Watersource3*	5.809**	2.468	4.390**	2.288	0.292**	0.138	0.180	0.145	− 0.025	0.072	− 0.033	0.028
*Off-farm*	− 0.511	2.038	− 0.517	2.288	0.119	0.122	− 0.007	0.137	− 0.005	0.063	− 0.003	0.095
*TLU (log)*	0.789***	0.061	0.655***	0.102	0.053***	0.004	0.053***	0.008	0.014**	0.007	0.019***	0.006
*Number of crops*	0.398	0.284	0.454***	0.088	0.080***	0.024	0.073***	0.015	0.018	0.017	0.039**	0.016
*Commercialization rate*	0.079***	0.003	0.072***	0.008	0.008***	0.001	0.007***	0.001	− 0.000	0.002	− 0.001	0.001
*Fertilizer*	− 0.378	0.557	0.883	0.605	0.022	0.033	0.089**	0.036	− 0.172***	0.058	− 0.077	0.050
*Pesticides*	2.300***	0.406	2.389***	0.936	0.171***	0.028	0.237***	0.069	0.036	0.061	0.015	0.052
*Price index deflated*	2.431***	0.413	2.301***	0.598	0.135***	0.011	0.199***	0.043	− 0.817***	0.057	− 0.570***	0.090
*Off-farm resid*	1.470	0.968	1.270	0.977	0.040	0.054	0.126***	0.049	0.023	0.015	0.036	0.041
*District FE*		Yes		Yes								
*_Cons*	38.107***	8.070	39.843***	9.512	5.031***	0.350	4.766***	0.630	8.512***	0.462	6.060***	0.697
*Sigma_1*	14.938***	0.075			1.253***	0.015			0.983***	0.048		
*Sigma_2*			14.634***	0.096			1.291***	0.015			0.865***	0.040
*Rho_1*	− 0.021	0.057			− 0.006	0.052			− 0.091**	0.040		
*Rho_2*			0.002	0.037			− 0.020	0.057			− 0.025	0.038

***p < 0.01, **p < 0.05, *p < 0.1

**Table 8:** Drivers of food and nutrition security outcomes (HAZ, FES, and BMI)

	BMI				HAZ				FES			
	Participants		Non- participants		Participants		Non- participants		Participants		Non- participants	
Variable	Coef.	Robust Std. Err.	Coef.	Robust Std. Err.	Coef.	Robust Std. Err.	Coef.	Robust Std. Err.	Coef.	Robust Std. Err.	Coef.	Robust Std. Err.
*Hh_size*	0.124***	0.045	0.056	0.053	− 0.006	0.011	− 0.013	0.025	− 0.007***	0.001	− 0.002	0.002
*Gender_head*	0.171*	0.097	− 0.018	0.162	− 0.107	0.083	− 0.010	0.044	0.013	0.010	− 0.001	0.004
*Age_head*	− 0.013***	0.004	− 0.016***	0.003	0.006***	0.002	0.001	0.002	− 0.001***	0.000	− 0.001***	0.000
*Education*	0.223***	0.040	0.155***	0.059	0.138***	0.021	0.129***	0.045	− 0.030***	0.004	− 0.028***	0.005
*Access_irrigation*	0.203	0.174	− 0.531	0.528	0.059	0.051	0.060	0.128	− 0.007	0.005	− 0.004	0.013
*Landcat2*	− 0.333	0.263	− 1.100***	0.148	− 0.096	0.111	− 0.134	0.116	0.019**	0.009	0.043***	0.015
*Landcat3*	− 0.276**	0.138	− 1.202***	0.210	− 0.165***	0.055	− 0.058	0.078	0.009	0.012	0.005	0.016
*Landcat4*	− 0.325	0.309	− 1.230***	0.278	− 0.200***	0.055	0.019	0.086	− 0.008	0.009	− 0.001	0.013
*Landcat5*	− 0.377**	0.169	− 0.881***	0.225	− 0.135**	0.057	− 0.022	0.135	− 0.033**	0.014	− 0.046**	0.022
*Landcat6*	− 0.447*	0.235	− 0.314	0.380	− 0.133**	0.056	0.234***	0.055	− 0.060***	0.014	− 0.059**	0.026
*Landcat7*	0.111	0.299	− 0.208	0.314	0.190	0.217	0.403**	0.162	− 0.077***	0.011	− 0.117**	0.048
*Marketcat1*	0.132	0.174	− 0.040	0.269	0.205***	0.070	0.107	0.084	− 0.008	0.018	− 0.043***	0.012
*Marketcat2*	0.132	0.191	0.093	0.181	0.048	0.048	0.121	0.079	− 0.009	0.006	− 0.012	0.008
*Marketcat3*	− 0.083	0.192	0.059	0.134	0.010	0.077	0.169**	0.072	− 0.003	0.006	− 0.016***	0.007
*Watersource1*	0.102	0.122	0.274**	0.137	− 0.204***	0.073	− 0.157	0.228	0.033***	0.010	0.010	0.015
*Watersource2*	0.425**	0.194	0.257**	0.128	− 0.177**	0.072	− 0.126	0.188	0.039***	0.011	0.020	0.016
*Off_farm_activities*	0.110	0.639	− 0.452	0.456	− 0.412	0.298	− 0.086	0.216	0.036*	0.022	0.030	0.020
*Log_TLU*	0.035*	0.019	− 0.006	0.041	0.015***	0.006	− 0.007	0.011	− 0.011	0.001	− 0.008**	0.001
*Number_crops*	− 0.014	0.069	0.018	0.037	0.015	0.023	0.015	0.023	− 0.005	0.003	− 0.012***	0.003
*Commercialization_rate*	0.004**	0.002	0.0001	0.002	0.002**	0.001	0.002	0.002	− 0.002***	0.000	− 0.002***	0.000
*Fertilizer*	0.064	0.101	0.296***	0.092	− 0.004	0.050	0.133***	0.024	− 0.027***	0.007	− 0.017	0.011
*Pesticides*	0.037	0.107	0.222	0.181	0.050	0.077	0.111***	0.031	− 0.029***	0.010	− 0.044**	0.019
*Price_index_deflated*	− 0.300***	0.049	0.010	0.180	− 0.054	0.050	− 0.077	0.051	0.013***	0.004	0.002	0.004
*Off_farmresid*	− 0.017	0.180	0.194	0.133	0.161	0.112	0.057	0.071	− 0.009	0.008	− 0.008	0.010
*Dist_code*	− 0.004***	0.001	− 0.003***	0.001	0.001**	0.000	0.001***	0.000	− 0.000**	0.000	0.000	0.000
*Constant*	25.746***	0.612	24.423***	1.959	− 1.427***	0.394	− 1.967***	0.159	0.494***	0.045	0.526***	0.056
*Sigma_1*	3.054***	0.097			1.354***	0.053			0.215***	0.027		
*Sigma_2*			3.123***	0.095			1.289***	0.032			0.218***	0.028
*Rho_1*	0.109**	0.081			− 0.027	0.062			− 0.120***	0.060		
*Rho_2*			− 0.037	0.056			− 0.022	0.070			− 0.009	0.026

*p < 0.1; **p < 0.05; ***p < 0.01

**Table 9:** Falsification Tests for validity of identifying Instruments in the ESR model estimation

Instrument	Garden	DDS	CDDS	FCS	BMI	HAZ	FES
Mean_age	2.721*** (0.089)	0.019 (0.071)	.038 (0.053)	0.837) (0.867)	0–.113 (0.244)	0.109 (0.105)	− .005 (0.012)
Mean_garden	2.672*** (0.092)	0.127 (0.071)	0.027 (0.051)	0.329) (0.858)	0.199 (0.284)	0–.095 (0.096)	.023 (0.011)
Cooperative	0.189*** (0.023)	0.610 (0.332)	5.270* (2.470)	0.641 (0.421)	0.413** (0.169)	.002 (0.007)	− .001 (0.001)
Constant	− 3.299*** (0.126)	19.948** (8.861)	6.768 (11.900)	892.56*** (142.063)	− 401.76*** ( 88.57)	− 4.843 (15.781)	2.65 (1.939)
Wald tests on instrument	χ^2^ = 5927.28*** (0.000)	F = 2.59 (0.0748)	F = 1.77 (0.1706)	F = 2.30 (0.1001)	F = 2.21 0.084)	1.41 (0.211)	F = 1.65 (0.066)

*p < 0.1; **p < 0.05; ***p < 0.01

## Footnotes

1 The 2018 Rwanda CFSVA is available online at: http://www.wfp.org/food-security and www.statistics.gov.rw

2 The specific categories are land size in hectare (ha) (0: no land, 1: 0–0.1, 2: 0.1–0.19, 3: 0.2–0.49, 4: 0.5–0.99, 5: 1–1.99, 6: < 2); market distance (0: in the village, 1: less than 60 min, 2: 60–120 min, 3: > 120 min), time to water source (0: less than 30 min, 2: 30–60 min, 3: > 60 min).

3 Instead of using crop level value which can be different among the main crops, we used the average sales share of the top three crops of the household. We employed a good number of other controls that could mitigate the potential inadequacy of this mersure of commercialization (market distance, location in rural/urban).

4 The details on the ESR approach are documented by Lokshin and Sajaia (2004).

5 The inclusion of the residuals *Off-farmresid* serves as control function and results in consistent estimate of the potentially endogenous explanatory variable (Wooldridge, 2015).

6 The Crop Intensification Program is a land consolidation program aimed at enhancing agricultural productivity and food security, https://www.files.ethz.ch/isn/128618/5712.pdf.
