# Supplementary material for: Heterogeneous impacts of home-gardening on household food and nutrition security in Rwanda
Source: Food Secur. 2023 Feb 18;15(3):731–50. doi: 10.1007/s12571-023-01344-w (PMC9938516; doi:10.1007/s12571-023-01344-w)
Supplement: Supplementary file 1 — Supplementary file1 (DOCX 27 KB) [file 12571_2023_1344_MOESM1_ESM.docx]

**Heterogeneous Impacts of Home-Gardening on Household Food and Nutrition Security in Rwanda**

Gazali Issahaku^1,2^, Lukas Kornher^1^, Abu Hayat Md. Saiful Islam^1,3^, Awal Abdul-Rahaman*^4^

^1^ Center for Development Research, University of Bonn, Bonn, Germany. Email: [lkornher@uni-bonn.de](mailto:lkornher@uni-bonn.de)

^2^Department of Food Security and Climate Change, University for Development Studies, Tamale, Ghana. Email: [igazali@uds.edu.gh](mailto:igazali@uds.edu.gh)

^3^Department of Agricultural Economics, Bangladesh Agricultural University, Mymensingh, Bangladesh. Email: [saiful_bau_econ@yahoo.com](mailto:saiful_bau_econ@yahoo.com)

^4^Department of Agribusiness, University for Development Studies, Tamale, Ghana

*Corresponding Author Email: [awalrahaman@uds.edu.gh](mailto:awalrahaman@uds.edu.gh)

**Running Head:** Home-Gardening and Household Food and Nutrition Security

**Abstract**

This study examines farmers’ decisions to engage in subsistence home-gardening and its impact on food and nutrition security among farm households in Rwanda under diverse conditions. The study uses a nationally representative dataset from Rwanda from 2012, 2015, and 2018. We employ an endogenous switching regression model to jointly estimate the drivers of home-gardening participation decisions and food and nutrition security outcomes, whiles accounting for selection bias from observable and unobservable factors. We also estimate the treatment effects of home-gardening participation on dietary diversity, food consumption score, and anthropometric markers of women and children. The treatment effects are calculated at sample means and connected to market-related variables, such as land ownership, commercialization extent, and market distance. We find that having a home-garden is linked to improved dietary diversity and better nutritional outcomes. If households have restricted access to land and reside further away from marketplaces, the benefits are larger. In contrast, the benefits of home-gardening are positive and significant regardless of the level of commercialization of production. We also discover that family size, gender, education, access to land, and livestock ownership are statistically significant drivers of home-gardening participation in Rwanda. However, the amount of commercialization did not affect a household’s decision to participate in home-gardening.

**Keywords**: Home-gardening; Food and nutrition security; Agricultural commercialization; Impact assessment; Rwanda.
