# Supplementary material for: Heterogeneous impacts of home-gardening on household food and nutrition security in Rwanda
Source: Food Secur. 2023 Feb 18;15(3):731–50. doi: 10.1007/s12571-023-01344-w (PMC9938516; doi:10.1007/s12571-023-01344-w)
Supplement: Supplementary file 2 — Supplementary file2 (DOCX 133 KB) [file 12571_2023_1344_MOESM2_ESM.docx]

**UNIVERSITY FOR DEVELOPMENT STUDIES**

**Faculty of Agriculture, Food and Consumer Sciences**

***Department of Agribusiness***

Tel: 071-22078/26633/26634	P. O. Box TL 1882

Fax: 071-22080/23957/23675	Nyankpala Campus

Mobile: (+233) 204024398	Tamale, Ghana

Email: awalrahaman@uds.edu.gh

Our Ref: P02.2174	27^th^ December, 2022

Your Ref:

The Editor

Food Security

Dear Editor,

Thank you for your letter dated December 27, 2022. My co-authors and I are glad to learn from you that we can revise the manuscript by addressing the minor comments raised by the reviewers and then re-submit same for publication. Attached please find a copy of “**Heterogeneous Impacts of Home-Gardening on Household Food and Nutrition Security in Rwanda**”, which we have revised and now resubmit for for publication in ***Food Security****.*

The minor comment has been very helpful in further improving the manuscript for re-consideration. We have revised the manuscript in line with the reviewer’s comments attached. Without necessarily repeating the responses to the reviewer, let me briefly summarize for you the changes we have made to this manuscript.

(1) In the revised manuscript, Bahta et al. (2018) and Depenbusch et al. (2021) have now been appropriately captured.
(2) We have also carefully read the entire manuscript and made the necessary correction/changes.

Once again, we thank you very much for supporting our manuscript. My co-authors and I look forward to hearing from you in due course. Thank you.

Sincerely yours,

Awal Abdul-Rahaman (Ph.D)
